# A Mathematical Model of Immune-System-Melanoma Competition

**DOI:** 10.1155/2012/850754

**Published:** 2012-06-03

**Authors:** Marzio Pennisi

**Affiliations:** Department of Mathematics & Computer Science, University of Catania, V.le A Doria 6, 95125 Catania, Italy

## Abstract

We present a mathematical model developed to reproduce the immune response entitled with the combined administration of activated OT1 cytotoxic T lymphocytes (CTLs) and Anti-CD137 monoclonal antibodies. The treatment is directed against melanoma in B16 OVA mouse models exposed to a specific immunotherapy strategy. We model two compartments: the injection point compartment where the treatment is administered and the skin compartment where melanoma tumor cells proliferate. To model the migration of OT1 CTLs and antibodies from the injection to the skin compartment, we use delay differential equations (DDEs). The outcomes of the mathematical model are in good agreement with the in vivo results. Moreover, sensitivity analysis of the mathematical model underlines the key role of OT1 CTLs and suggests that a possible reduction of the number of injected antibodies should not affect substantially the treatment efficacy.

## 1. Introduction

 Melanoma is a malignant tumor caused by the mutation of melanocytes, that is, the cells that produce the melanin and are responsible of the color of the skin. Despite intensive research, melanoma still represents one of the most aggressive malignant cancers [1]. Many experimental approaches are now focused on targeting cytotoxic T lymphocytes (CTLs) against cancer. A common strategy to enable CTL efficacy against tumor is to activate naïve CTLs in vitro through the use of cells engineered to present the tumor antigen, and to reinject them in the host. However, even if activated CTLs are able to infiltrate into tumor masses, in most cases they remain unable to contrast cancer growth [2]. As experimental evidence suggests, tumor-infiltrating lymphocytes are rendered ineffective by coinhibitory molecules expressed by tumor and stroma cells surfaces [3].

In order to gain complete rejection of tumors, injection and stimulation of CTLs is not sufficient and should be, therefore, coupled with complementary measures voted at boosting CTLs migration inside tumor masses, and conjugation and killing of target cells [4–6]. One way of boosting CTLs actions is represented by stimulation through the binding of costimulatory proteins expressed on CTLs surface. Among possible surface proteins, Anti-CD137, also known as 4-1BB, represents a valuable target. This protein is expressed by multiple IS cells such as activated T, NK, B-lymphocytes, dendritic cells and also by tumor endothelium cells [7]. Its natural ligand (CD137L) can be found on activated antigen-presenting cells surface [8].

The combined administration of monoclonal antibodies specifically targeted to bind Anti-CD137 proteins and in vitro activated-OT1 CTLs was demonstrated to be able to prevent the melanoma formation in B16-OVA mouse models [7]. Moreover, the combined treatment avoided appearing of undesired side effects like the hepatotoxicity, observed only under anti-CD137 only high-dosage treatment [9]. The IS stimulation mechanisms of Anti-CD137 immunostimulatory monoclonal antibodies are multilayered and include the improving of cytotoxicity, duplication rates, and chemotaxis sensitivity of activated-OT1 CTLs [6, 10–12].

To reproduce the dynamics of this biological process, a delay differential-equation-(DDE-) based model has been developed. The model reproduces two different compartments: the injection point compartment, where both antibodies and OT1 cells are injected and the skin compartment where melanoma develops.

## 2. Biological Background

The in vivo experiment is carried on B16-OVA mice, mice transduced with the chicken ovalbumin gene. The ovalbumin is used as a model tumor antigen. B16 melanoma cell line was derived from an aggressive spontaneous melanoma in pure C57BL6, and B16F10 was derived as a clonal variant from a lung metastasis of this cell line. In tumor immunology, these variants of melanoma are considered poorly immunogenic in the sense that immune-mediated rejections or growth retardations are difficult to achieve.

The experimental setup is oriented to model therapeutic synergy between anti-CD137 monoclonal antibodies and adoptive T cell therapy in melanoma. B16-OVA is a poorly immunogenic murine tumor. The treatment protocol includes a single injection of anti-CD137 mAb and adoptive T cell transfer of OVA-specific TCR-transgenic CD8 CTLs.

In vivo experiments have been executed by Professor Melero and coworkers at the University of Navarra [13]. Mice are divided in five different groups; all groups are composed by five individuals. Each group is treated with a different treatment: Untreated (control) mice, mice treated with naïve OT1 CTLs, mice treated with naïve OT1 CTLs and Anti-CD137 monoclonal antibodies, and mice treated with in vitro activated OT1 CTLs, mice treated with Anti-CD137 monoclonal antibodies, mice treated with in vitro activated OT1 CTLs and Anti-CD137 monoclonal antibodies. The experiment runs for 30 days. At day 0, all B16-OVA mice receive one injection of melanoma malignant cells. The therapeutic treatment used during in vivo experiments is composed by one single boost, and it is administered at day 3. Melanoma surface measurements (mm^2^) are taken at given times for each treatment and are used to estimate the efficacy of each vaccination strategy. We note here that in order to compare in vivo and in silico results we computed the estimated mean surfaces entitled with the use of each treatment. Among the tested treatments, only the combined administration of activated OT1 CTLs and antibodies was able to show complete depletion of the tumor burden, whereas the other treatments remained almost ineffective [13].

## 3. The Model

**Figure 1:**
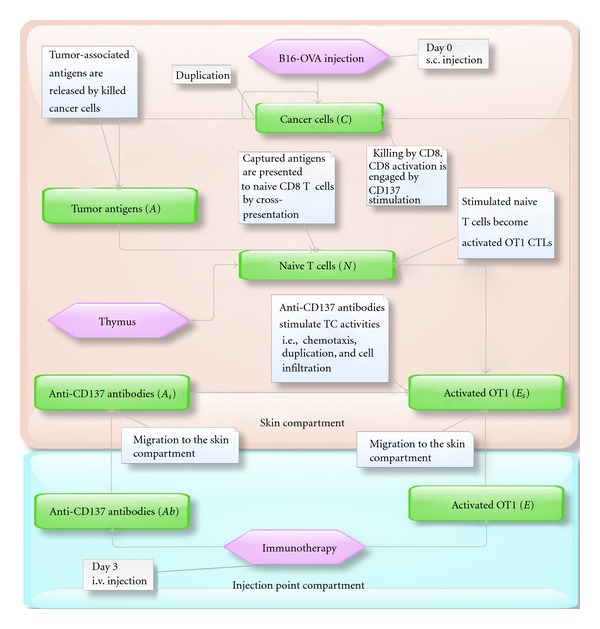
Conceptual model of the biological scenario. The model is composed by two compartments: the first compartment (bottom) where the treatment is administered, and the skin compartment (top) where the immune-system-melanoma competition occurs. Arrows are used to indicate the interaction between entities (i.e., the interaction between the antibodies and activated CTLs in the skin compartment), the change of a particular condition of an entity (i.e., the activation of naïve CTLs or the migration of antibodies from a compartment to another), the introduction and disappearing of entities (i.e., the production of newborn naïve CTLs by thymus or the disappearing of antigens that are presented to naïve CTLs). White boxes are used to better explain the meaning of the arrows. Both antibodies (*Ab*) and activated OT1 CTLs (*E*) migrate to the skin compartment. In the skin compartment, antibodies (*A*_*s*_) stimulate duplication and infiltration into tumor mass of activated OT1 CTLs (*E*_*s*_), which kill melanoma cells (*C*). Killed melanoma cells release antigens (*A*) that are captured by antigen presenting cells and presented to antigen-specific naïve OT1 CTLs (*N*). Naïve OT1 CTLs are then stimulated to become active CTLs (*E*_*s*_).

 We realized a model with two compartments in order to reproduce the dynamics of the process. The first compartment is represented by the injection point compartment where the treatment is administered, whereas the second one is represented by the skin compartment where melanoma tumor cells proliferate and where the cancer-IS competition occurs. To this end, a system of seven delay differential equations has been set up. The model takes into account the following entities: injected activated OT1 CTLs (*E*) and injected antibodies (*Ab*) for the injection point compartment; melanoma cells (*C*), tumor antigens (*A*), activated OT1 CTLs and antibodies that have reached the skin (*E*_*s*_ and *A*_*s*_) and naïve CTLs (*N*) for the skin compartment. It has also been assumed that Injected OT1 CTLs, and antibodies move from the injection point to the skin compartment only.  Figure 1 shows the conceptual model for the biological problem; model entities are listed in Table 1.

(1) Activated OT1 CTLs (injection point compartment):

(1) $$\begin{array}{cc} \frac{dE}{dt} & =K_{\text{in}}\left(t,p\right)-\alpha_{11}E-\alpha_{8}E \end{array}.$$

Equation (1) refers to first compartment and represents the time evolution of injected activated OT1 CTLs. In (1), *K*_in_(*t*, *p*) represents a known function that models the number of inoculated entities *r* at the scheduled injection time *t*. *E* cells migrate from the injection point compartment to the skin compartment with given rates (−*α*_11_ *E*) and are subject to natural death (−*α*_8_ *E*).

(2) Antibodies (injection point compartment):

(2) $$\begin{array}{cc} \frac{dAb}{dt} & =K_{\text{in}}\left(t,q\right)-\alpha_{11}Ab-\alpha_{10}Ab. \end{array}$$

Similary to (1), (2) refers to the first compartment and represents the time evolution of antibodies (*Ab*). *Ab* are injected at given times *t* and at given quantities *q*, according to the function *K*_in_(*t*, *q*), and can migrate and disappear from the system by natural degradation at given rates (−*α*_11_ *Ab* and −*α*_10_ *Ab*, resp.).

(3) Activated OT1 CTLs (skin compartment):

(3) $$\begin{array}{cc} \frac{dEs}{dt} & =\alpha_{7}\left[\frac{A_{s}}{A_{s}+k_{1}}\right]E_{s}+\alpha_{11}E\left(t-\tau \right)+\alpha_{6}NA-\alpha_{8}E_{s}. \end{array}$$

Activated OT1 CTLs that reach the skin compartment (*E*_*s*_) are modeled by (3). Antibodies in the skin compartment (*A*_*s*_) have multiple positive effects on activated OT1 CTLs dynamics (*E*_*s*_). One of these effects is represented by the ability of promoting *E*_*s*_ duplication. This is modeled through the Holling type II function *α*_7_[*A*_*s*_/(*A*_*s*_ + *k*_1_)]*E*_*s*_, where *α*_7_ is the maximum biological duplication rate of *E*_*s*_ and *k*_1_ is a tuned threshold. When the number of *A*_*s*_ is high enough, the term [*A*_*s*_/(*A*_*s*_ + *k*_1_)] tends towards 1, thus entitling maximum duplication rates for *E*_*s*_. The term *α*_11_ *E*(*t* − *τ*) is used to model migration of OT1 CTLs from the injection compartment to the skin compartment. We suppose here that migration from the first compartment (*E*) to the second one (*E*_*s*_) entitles a time delay of *τ* and occurs with a given rate *α*_11_.

The term *α*_6_ *NA* models the activation of naïve CTLs *N* thanks to the presence of antigens (*A*) released by killed cancer cells. The biological process that explains the presence of this term is summarized as follows. Antigenic sequences released by killed melanoma cells may be captured by antigen presenting cells (APC) such as macrophages and dendritic cells. These cells process the antigens and present them on their cellular surface to naïve CTLs (*N*). After this presentation process, naïve CTLs cells may be activated, and if some complementary biological steps are accomplished (i.e., stimulation by cytokines released by T helper cells), they can become able to kill tumor cells. This process is not modeled in depth since it involves the modeling of other entities that are not considered fundamental for the problem. The number of newly activated OT1 CTLs is instead directly estimated on the basis of the quantity of released antigens. The last term (−*α*_8_ *E*_*s*_) is used to take into account natural death of OT1 CTLs.

(4) Melanoma cells:

(4) $$\begin{array}{cc} \frac{dC}{dt} & =\left(\alpha_{1}-\alpha_{2}\ln\left(C\right)\right)\cdot C-\alpha_{3}\left[\frac{A_{s}+k_{2}}{A_{s}+k_{3}}\right]E_{s}C. \end{array}$$

Equation (4) describes the melanoma cells (*C*) behavior in the skin compartment. The first term ((a_1_ − a_2_ln⁡(*C*)) · *C*) represents a Gompertzian growth [14], whereas the second term denotes killing of *C* by activated OT1 CTLs that are already in the skin compartment (*E*_*s*_). One of the most important actions accomplished by antibodies (*A*_*s*_) is to boost chemotaxis sensitivity of CTLs (*E*_*s*_) thus enabling better infiltration into the tumor mass. This translates into higher killing rates of melanoma cancer cells by activated CTLs (*E*_*s*_). This is modeled through the term −*α*_3_[(*A*_*s*_ + *k*_2_)/(*A*_*s*_ + *k*_3_)]*E*_*s*_ *C*, where *α*_3_ is the maximum killing rate of *C* by *E*_*s*_, and *k*_2_ and *k*_3_ (*k*_2_ ≪ *k*_3_) are tuned constants. When the number of *A*_*s*_ is high enough, the term [(*A*_*s*_ + *k*_2_)/(*A*_*s*_ + *k*_3_)] tends towards 1, thus entitling maximum killing rates for *E*_*s*_. In absence of antibodies, having *k*_2_ ≪ *k*_3_, the term translates into −*α*_3_ *k*_2_/*k*_3_ < *α*_3_, which involves lower killing rates.

(5) Antigens:

(5) $$\begin{array}{cc} \frac{dA}{dt} & =\alpha_{4}\left[\alpha_{3}\left[\frac{A_{s}+k_{2}}{A_{s}+k_{3}}\right]E_{s}C\right]-\alpha_{5}A-\alpha_{6}NA. \end{array}$$

With (5), we describe the tumor-associated antigen (*A*) dynamics. Antigens are released in the skin compartment by killed melanoma cells (*α*_4_ · [*α*_3_[(*A*_*s*_ + *k*_2_)/(*A*_*s*_ + *k*_3_)]*E*_*s*_ *C*]) and are subject to natural degradation (−*α*_5_ *A*). They can also be captured by APC, which will present the antigen to naïve CTLs. As already stated in (3), capturing of the antigen by APC is not modeled and the number of captured antigens is estimated on the basis of naïve CTLs (*N*) that are activated by APC (−*α*_6_ *NA*).

(6) Naïve CTLs:

(6) $$\begin{array}{cc} \frac{dN}{dt} & =h\left(M-N\right)-\alpha_{6}NA. \end{array}$$

Equation (6) models the behavior of naïve OT1 CTLs (*N*). It is supposed here that these cells are already present in the skin compartment. The term *h*(*M* − *N*) is used to model homeostasis. *M* is the number of circulating naïve CTLs under safe conditions given by the leukocyte formula. If switching of naïve CTLs to activated CTLs occurs, the number of naïve CTLs gets lower. As a consequence of that, the naïve population is repopulated with newborn cells and tends towards *M* at a rate *h*. The second term (−*α*_6_ *NA*) models the CTLs state changing from naïve to activated (*E*_*s*_), thanks to presentation of the antigen by APC.

(7) Antibodies (skin compartment):

(7) $$\begin{array}{cc} \frac{dA_{s}}{dt} & =\alpha_{11}Ab\left(t-\tau \right)-\alpha_{9}A_{s}E_{s}-\alpha_{10}A_{s}. \end{array}$$

Antibodies that have reached the skin compartment (*A*_*s*_) are modeled and described by (7). Antibodies in the skin compartment are supposed to be proportional to the number of antibodies in the injection point compartment (*Ab*) with a proportionality constant *α*_11_ and a time delay of *τ*. They also disappear by stimulating OT1 cells activities and are subject to a natural degradation (−*α*_9_ *A*_*s*_ *E*_*s*_ and −*α*_10_ *A*_*s*_).

According to the considered cell populations and interactions, the mathematical model can be then represented by the following system of seven nonlinear delay differential equations:

(8) $$\begin{array}{cc} & \frac{dE}{dt}=K_{\text{in}}\left(t,p\right)-\alpha_{11}E-\alpha_{8}E,\\ & \frac{dAb}{dt}=K_{\text{in}}\left(t,q\right)-\alpha_{11}Ab-\alpha_{10}Ab,\\ & \frac{dE_{s}}{dt}=\alpha_{7}\left[\frac{A_{s}}{A_{s}+k_{1}}\right]E_{s}+\alpha_{11}E\left(t-\tau \right)+\alpha_{6}NA-\alpha_{8}E_{s},\\ & \frac{dA_{s}}{dt}=\alpha_{11}Ab\left(t-\tau \right)-\alpha_{9}A_{s}E_{s}-\alpha_{10}A_{s},\\ & \frac{dN}{dt}=h\left(M-N\right)-\alpha_{6}NA,\\ & \frac{dC}{dt}=\left(\alpha_{1}-\alpha_{2}\ln\left(C\right)\right)\cdot C-\alpha_{3}\left[\frac{A_{s}+k_{2}}{A_{s}+k_{3}}\right]E_{s}C,\\ & \frac{dA}{dt}=\alpha_{4}\left[\alpha_{3}\left[\frac{A_{s}+k_{2}}{A_{s}+k_{3}}\right]E_{s}C\right]-\alpha_{5}\text{A}-\alpha_{6}NA. \end{array}$$

Since we consider mainly populations that appear in the system as a consequence of treatment administrations (except for melanoma and naïve CTLs), the following Cauchy initial conditions have been set for the equations:

(9) $$\begin{array}{c} \begin{array}{c} E\left(0\right)=0,\;Ab\left(0\right)=0,\;E_{s}\left(0\right)=0,\;A_{s}\left(0\right)=0,\;A\left(0\right)=0,\\ N\left(0\right)=M,\;C\left(0\right)=C_{0}. \end{array} \end{array}$$

The physical time-step Δ(*t*) has been chosen equal to 8 hours, and the integration time has been then computed up to 10^2^ · Δ(*t*) ≈ 33 days. The reason of this choice is biological, and it is given by the fact that in the in vivo experiment it is not possible to observe relevant biological phenomena in smaller time intervals. In particular, the minimum time required for cell division, which represents one of the most important biological phenomena, is usually not lower than 6–8 hours [15]. This may be not true in other in vivo setups, such as in case of allergies, where the time scale varies from seconds to minutes.

Some parameters appearing in the equations have been estimated from the literature (see Table 2), and from measurements made during the in vivo experiment.

In particular, melanoma growth rates have been estimated from diameter measurements made at different times in melanomas in five untreated mice. Melanomas have two growth phases, radial and vertical [16]. The first phase is represented by a radial growth, in which malignant cells grow in a radial fashion in the epidermis. At later stages, most melanomas progress to the vertical growth phase, in which the malignant cells invade the dermis and develop the ability to metastasize. In this case, we supposed that only the radial growth phase is involved. By supposing a disk-shaped layout for the melanoma and having knowledge of the mean diameter of melanoma cancer cells, the number of cancer cells (in the observed melanomas) has been estimated for all the measurements made. This data has been used with a curve fitting procedure to estimate the unknown parameters needed to model the cancer growth kinetics under the hypothesis of a Gompertzian growth for the tumor, used successfully in our previous experience [13, 17, 18]. The Gompertz law, which is commonly considered suitable for describing populations growths, uses two factors: a constant growth term and a shrink term that increases in time and is related to antiangiogenic factors, giving as a result a sigmoid shape to the curve. The initial number of injected cancer cells *C*_0_, the number of injected OT1 CTLs and antibodies (*p* and *q*), and the number of antigen-specific naïve OT1 CTLs under safe conditions (*M*) have been chosen into reasonable ranges given by in vivo measurements.

Remaining parameters have been chosen in plausible biological ranges in such a way to reproduce the set of experimental data for the activated OT1 CTLs + Anti-CD37 Ab combined treatment, and counterchecked against the other vaccine scenarios taken into account (Table 2).

## 4. Results

To reproduce the dynamics of the model, we took into account four treatments that have been tested in vivo: untreated (control), in vitro activated OT1 CTLs, Anti-CD137 monoclonal antibodies, and in vitro activated OT1 CTLs + Anti-CD137 monoclonal antibodies.

Among such treatments, only the last one (treated with activated CTLs + Anti-CD137 monoclonal antibodies) showed complete eradication of the tumor burden, whereas the other treatments remained almost ineffective.

**Figure 2:**
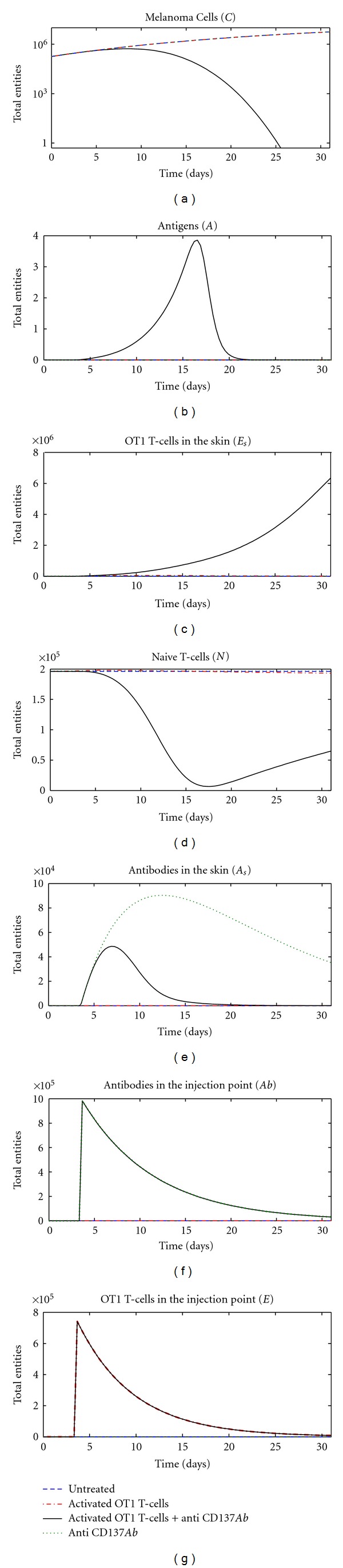
System behavior obtained with the use of different vaccination protocols: untreated (blue dashed line), activated OT1 CTLs (red dot-dashed lines), Anti-CD137 monoclonal antibodies (green dotted lines), and activated OT1 CTLs + Anti-CD137 monoclonal antibodies (black solid lines). From left to right, top to bottom: melanoma cells (*C*), tumor antigens (*A*), activated OT1 CTLs in the skin (*E*_*s*_), naïve CTLs (*N*), antibodies in the skin (*A*_*s*_), antibodies in the injection point (*Ab*), and activated OT1 CTLs in the injection point (*E*).

Entities behaviors for all the analyzed treatments are shown in Figure 2. In absence of therapy (blue dashed line), there is no induced immune response and thus the number of melanoma cells grows without any intervention from IS cells, whose plots remain flat.

The same scenario arises when the Anti-CD137 monoclonal antibodies treatment is administered (see Figure 2, green dotted lines). The mechanism that triggers the IS response is driven by the presence of activated OT1 CTLs that can kill melanoma cells, which release antigens able to further stimulate the IS response.

This fact can be partially seen if the treatment based upon the administration of in vitro activated OT1 CTLs is used (Figure 2, red dot-dashed lines). The *E*_*s*_ plots show some evidence of activated OT1 CTLs in the skin. These cells are able to kill melanoma cells (*C*), which release antigens (*A*) that are captured by APCs and then used to promote the differentiation of newborn naïve CTLs (*N*) to activated OT1 CTLs (*E*_*s*_). However, in absence of Anti-CD137 antibodies, which promote both duplication and infiltration into tumor masses of activated OT1 CTLs, recruitment of newborn naïve CTLs is too bland and tardy to stimulate an IS response able to stop the melanoma.

Only the combined action of activated CTLs + Anti-CD137 monoclonal antibodies is able to to contrast the melanoma growth (see Figure 2, black solid line). In this case, both Anti-CD137 antibodies (*Ab*) and activated OT1 CTLs (*E*) are injected at day three and then migrate to the skin compartment. Activated OT1 CTLs (*E*_*s*_) are stimulated to duplicate and to infiltrate into tumor masses thanks to the presence of antibodies in the skin compartment (*A*_*s*_). As a consequence of this, many more melanoma cells (*C*) are killed, and copious number of antigens (*A*) is released, thus promoting the activation of naïve CTLs (*N*) to activated OT1 CTLs (*E*_*s*_), which further act against melanoma.

To qualitatively compare the model results with the in vivo data, we estimated (using the number of cancer cells) the melanoma surface given by the mathematical model under the same assumptions made for estimating the Gompertz growth parameters. We then compared the time evolution of the melanoma surface given by the mathematical model with the mean surface observed in the in vivo experiment for the following cases: the untreated (control) case and the activated CTLs + Anti-CD137 monoclonal antibodies combined treatment case. The other cases are not presented since they give back the same scenario observed in the untreated case for both the experiments.

**Figure 3:**
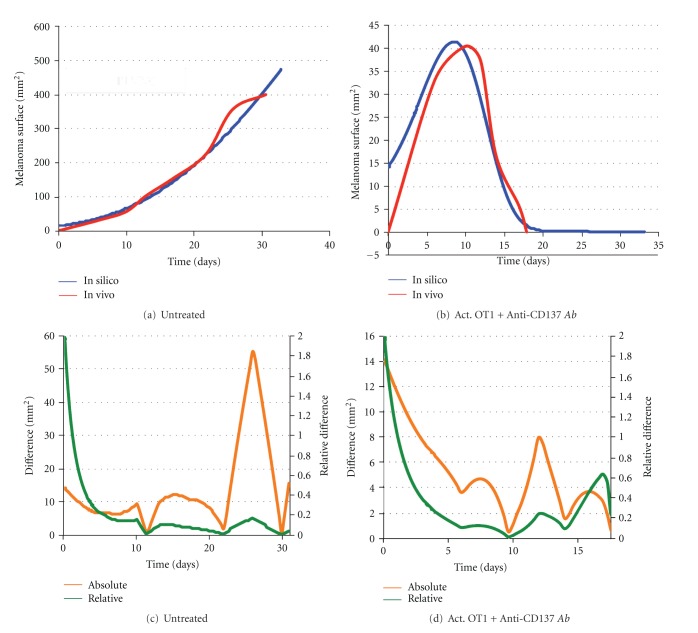
Comparison of melanoma surface (mm^2^) behavior over time between mean in vivo measurements (red lines) and simulations (blue lines). (a) Comparison for the untreated case (no vaccination). (b) Comparison under the administration of the combined activated OT1 CTLs + Anti-CD137 monoclonal antibodies treatment. In silico melanoma surfaces have been estimated from the total number of melanoma cells by assuming only radial growth and a disk-shaped layout for the melanoma. (c) Absolute (orange line) and relative (green line) differences between in vivo and in silico measurements for the untreated case (no vaccination). (d) Absolute (orange line) and relative (green line) differences between in vivo and in silico measurements under the administration of the combined activated OT1 CTLs + Anti-CD137 monoclonal antibodies treatment.

Comparison for the untreated case is shown in Figure 3(a). As expected, since we only observe the melanoma growth, the mathematical model perfectly reproduces the in vivo setup. Comparison for the combined treatment case is shown in Figure 3(b). The mean in vivo melanoma behavior is well reproduced by the mathematical model that is able to quantitatively and qualitatively represent the shape of the curve observed in the in vivo experiments. In Figures 3(c) and 3(d), we show absolute and relative differences between in silico and in vivo measurements for the untreated and activated OT1 CTLs + Anti-CD137 combined treatments, respectively. Relative differences are computed using the following metric: *d*(*x*, *y*) = |*x* − *y* | /(1/2(|*x* | +|*y*|)).

It must be noted here that the initial gap between the mathematical model and the in vivo experiment measurements visible in Figures 3(b) and 3(d) is mainly due to the different nature of measurements. In the simulations, the total number of cells is always known and it is used to estimate the melanoma surface. In the in vivo experiment, melanoma surface is instead measured on mice skin. Even if the injection of tumor cells is done at time 0, melanoma needs some time to arise and become visible even if the melanoma cells are all already present in mice.

## 5. Sensitivity Analysis

To understand how the system varies under different parameter values, it is important to analyze the sensitivity of the model to variation of parameters. Classical sensitivity analysis is done usually by varying a given parameter in reasonable ranges and keeping the other ones fixed. The results obtained are obviously dependent on the values of the fixed parameters, and different sets of values may entitle completely different results.

In order to overcome the limits of classical sensitivity analysis, many techniques have been developed. One of these is represented by Partial rank correlated coefficients (PRCC) [23], a statistical sensitivity analysis technique, which computes a partial correlation on rank-transformed data between input and output values, represented in this case by the model parameters (input) and the model entities behaviors (output).

It important to note here that the obtained correlation indexes do not depend on a given set of parameters, and it is therefore possible to estimate how the variations of a parameter may influence the results of the model, no matter what the value of the other parameters is. PRCC returned values varying in [−1,1] and estimated the correlation between input and output parameters. A value near 1 suggests a high (linear) positive correlation, whereas a value near −1 indicates a negative correlation. Values around 0 usually indicate little or no correlation.

Using this methodology in conjunction with the Latin Hypercube Sampling (LHS), which is used to sample the parameters' space, we analyze the effects of the input parameters most influencing the growth of cancer cells (*C*). We plot PRCCs versus the experiment time for the most important parameters to see how the sensitivity of parameters changes as the system dynamics progresses.

### 5.1. Impact Variation of the Treatment Quantities *p* and *q* on the Number of Melanoma Cells

**Figure 4:**
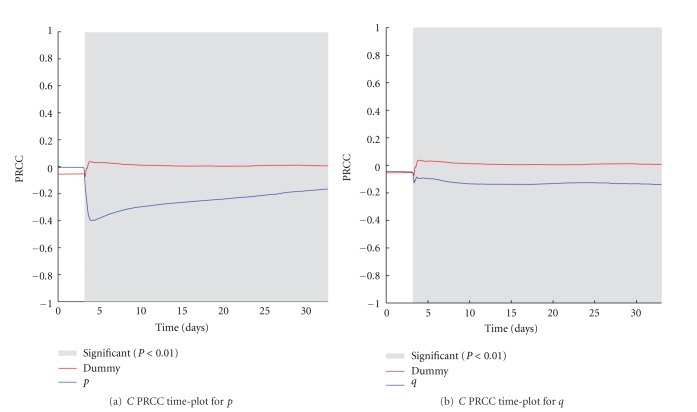
*p* (a) and *q* (b) PRCC plots computed on *C*. PRCC values are calculated with respect to the number of melanoma cells (*C*), plotted over time (blue lines). PRCC time plot of Dummy parameter (red lines) is shown for comparison. Greyed areas represent the plot portions where correlation is significant (*P* < 0.01).

 The two parameters *p* and *q* refer to the number of activated OT1 effector cells (*E*) and Anti-CD137 antibodies (*Ab*) injected as a single boost treatment against the melanoma, according to the in vivo experiment. In Figure 4, we show the PRCC time plots for the two parameters. As expected, just after the injection of the treatment, both the two parameters show a negative correlation with the number of cancer cells. It is worth to note that *p* shows good correlation in particular in the first ten days, whereas a weaker and almost constant correlation is related to *q*. This may suggest that the effects of the treatment are mainly driven by the OT1 effector cells and, even if antibodies are needed to obtain protection (as discussed earlier and shown in Figure 2), a reduction in their quantity may entitle similar treatment effectiveness.

### 5.2. Impact of Naïve OT1 CTLs Initial Number (*M*) and Recovery Rate (*h*) on the Number of Cancer Cells

**Figure 5:**
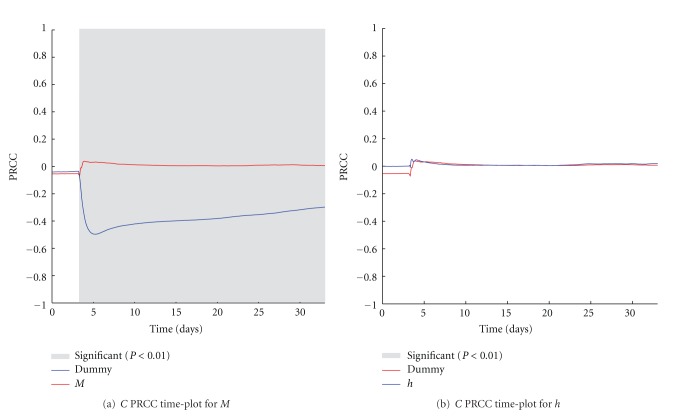
*M* (a) and *h* (b) PRCC plots computed on *C*. PRCC values are calculated with respect to the number of melanoma cells (*C*), plotted over time (blue lines). PRCC time plot of Dummy parameter (red lines) is shown for comparison. Greyed areas represent the plot portions where correlation is significant (*P* < 0.01).

 The *M* parameter indicates the initial number of antigen-specific naïve CTLs in the host, and the *h* parameter represents the rate of newborn naïve CTLs generated by thymus selection. The *M* parameter negatively correlates over time with the number of melanoma cells (Figure 5(a)), thus confirming that the action of naïve OT1 T cells induced by the treatment administration is fundamental for treatment effectiveness. Surprisingly it seems that the variation of the rate of introduced newborn naïve CTLs does not influence the number of melanoma cells, since no correlation is shown over time (see Figure 5(b)).

### 5.3. Impact of Activated OT1-T-Related Parameters on the Number of Cancer Cells

**Figure 6:**
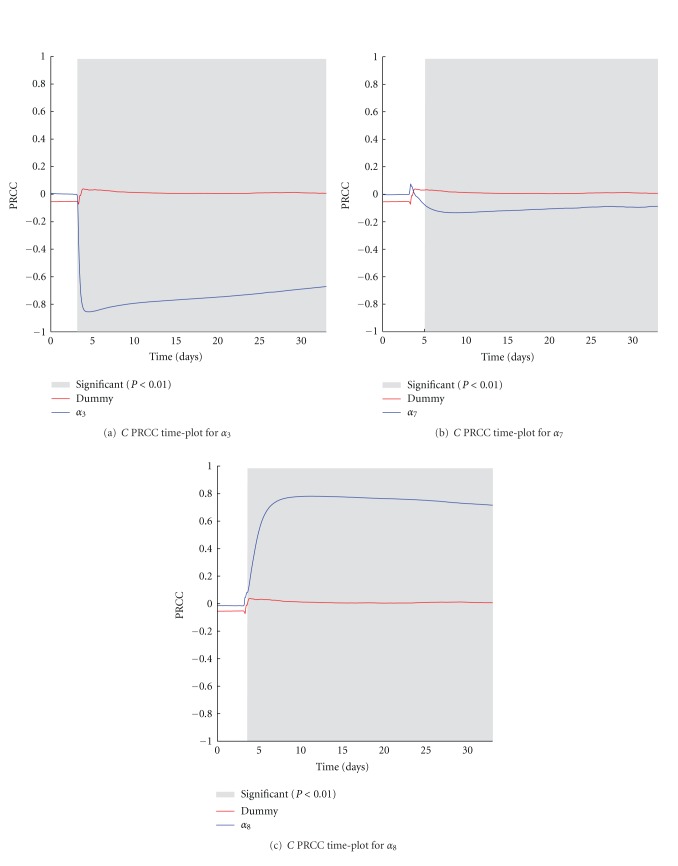
*α*_3_ (a), *α*_7_ (b), and *α*_8_ (c) PRCC plots computed on *C*. PRCC values are calculated with respect to the number of melanoma cells (*C*), plotted over time (blue lines). PRCC time plot of Dummy parameter (red lines) is shown for comparison. Greyed areas represent the plot portions where correlation is significant (*P* < 0.01).

 The activated OT1 CTLs in the skin (*E*_*s*_) represent the number of entities that are directly able to kill melanoma tumor cells. It is, therefore, trivial to see that *α*_3_ and *α*_8_, which represent the rate of killed melanoma cells by activated OT1 effector cells and the half-life of OT1 effector cells, have a strong negative and positive correlation with the number of cancer cells, respectively, (see Figures 6(a) and 6(b)). A negative correlation is also present for *α*_7_, which represents the maximal duplication rate of (*E*_*s*_) (see Figure 6(c)). However, in this case the correlation remains weaker, thus suggesting how small variations on the cells duplication rates, which can be associated with individual diversity, do not influence considerably the total behavior of cancer cells.

### 5.4. Impact of Antibody-Related Parameters on the Number of Cancer Cells

**Figure 7:**
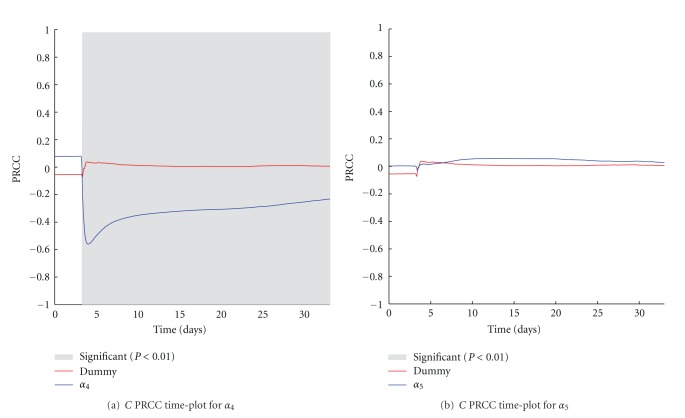
*α*_4_ (a) and *α*_5_ (b) PRCC plots computed on *C*. PRCC values are calculated with respect to the number of melanoma cells (*C*), plotted over time (blue lines). PRCC time plot of Dummy parameter (red lines) is shown for comparison. Greyed areas represent the plot portions where correlation is significant (*P* < 0.01).

 The *α*_4_ parameter is used to represent the rate of antigens released by killed *C*, whereas the *α*_5_ represents the specific antigen half-life. If the former has a strong negative PRCC correlation with the number of *C* (Figure 7(a)), particularly in the first period just after treatment administration, the latter interestingly seems to be not correlated with the number of *C* (Figure 7(b)).

### 5.5. Impact of the Tumor Growth and Shrink Parameters (*α*_1_ and *α*_2_) on the Number of Cancer Cells

**Figure 8:**
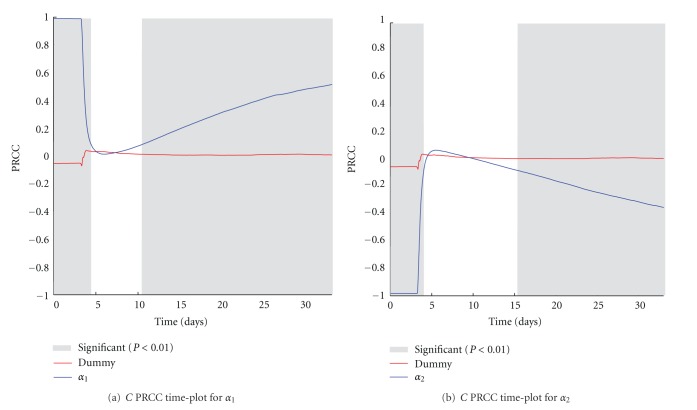
*α*_1_ (a) and *α*_2_ (b) PRCC plots computed on *C*. PRCC values are calculated with respect to the number of melanoma cells (*C*), plotted over time (blue lines). PRCC time plot of Dummy parameter (red lines) is shown for comparison. Greyed areas represent the plot portions where correlation is significant (*P* < 0.01).

 The *α*_1_ and *α*_2_ parameters represent the Gompertz growth parameters used to model the growth of melanoma. The range of variation for these two parameters has been set equal to the confidence range given by the curve fitting procedure used to estimate the parameters' values from in vivo measurements. In this way, we reasonably take into account the possible melanoma growths that may also be observed in the in vivo experiments. PRCC plots over time are represented in Figures 8(a) and 8(b). Even if positive and negative PRCC correlations are somewhat expected, there is a small time window just after the treatment injection time where no correlation occurs. This may be explained by the fact that variations of the melanoma growth rate do not influence substantially (at least initially) the effectiveness of the treatment.

## 6. Conclusions

We presented a mathematical model, which reproduces the immune response against B16-melanoma induced by the combined administration of activated OT1 CTLs and Anti-CD 137 immunostimulatory monoclonal antibodies. The model uses delay differential equations to reproduce the presence of two different compartments: the injection point compartment where the treatment is administered, and the skin compartment, where the melanoma-Immune system competition occurs.

The model proved to be able to coherently reproduce the in vivo experiment results obtained with four vaccination strategies (untreated, only activated OT1 CTLs, only monoclonal *Ab*, OT1 CTLs + monoclonal *Ab*). Moreover, the model is able to qualitatively and quantitatively reproduce the time dynamics of melanoma under the administration of the combined treatment. Results show that activated CTLs + Anti-CD137 monoclonal combined treatment acts in two ways: directly by activated OT1 CTLs that are able to kill melanoma and antibodies that boost CTLs activities and indirectly by promoting activation of naïve OT1 CTLs thanks to the releasing of melanoma cells antigens.

Among some useful findings, sensitivity analysis underlined the important role of activated OT1 cytotoxic treatment, suggesting that it would be in principle possible to obtain similar effectiveness lowering the number of administered antibodies, which, however, remain fundamental to gain effectiveness. Such kind of suggestions may be useful for optimizing treatment effectiveness and minimizing the risk of side effects.

Future work will be focused on studying analytically a simplified model without delay and in comparing the obtained results with Sim-B16, an agent-based model developed to reproduce the same in vivo experiment [13].

## Figures and Tables

**Table 1:** Model variables. Each variable describes the total number of the related entity in the associated compartment.

Variable	Description	compartment
*E*	Injected activated OT1 CTLs	injection point
*Ab*	Injected anti-CD137 antibodies	injection point
*C*	Melanoma tumor cells	skin
*A*	Tumor antigens	skin
*E*_*s*_	Activated OT1 CTLs	skin
*A*_*s*_	Injected anti-CD137 antibodies	skin
*N*	Naïve CTLs	skin

**Table 2:** Model parameters. The “in vivo” label refers to parameters that have been chosen according to in vivo measurements and observations. The “Estimated” label refers to free (unknown) parameters that have been tuned.

Param.	Description	Value (estimate)	Ref.
*α*_1_	*C* growth parameter (Gompertz)	0.2165 Δ(*t*)^−1^	in vivo
*α*_2_	*C* shrink parameter (Gompertz)	0.01269 Δ(*t*)^−1^	in vivo
*α*_3_	*C* max killing rate by *E*_*s*_	0.00000033 Δ(*t*)^−1^	Estimated
*α*_4_	*A* release rate by Killed *C*	0.21 Δ(*t*)^−1^	Estimated
*α*_5_	*A* natural degradation rate	ln(2)/9 Δ(*t*)^−1^	[19]
*α*_6_	Switch rate from *N* to *E*_*s*_ thanks to *A*	0.1 Δ(*t*)^−1^	Estimated
*α*_7_	*E*_*s*_ max duplication rate	0.095 Δ(*t*)^−1^	Estimated
*α*_8_	*E* and *E*_*s*_ nautral death rate	ln(2)/15 Δ(*t*)^−1^	[20]
*α*_9_	*Ab* death rate due to stimulation of *E*_*s*_	0.000001 Δ(*t*)^−1^	Estimated
*α*_10_	*Ab* and *A*_*s*_ natural degradation rate	ln(2)/21 Δ(*t*)^−1^	[21, 22]
*α*_11_	migration rate from injection to skin compartment	0.009 Δ(*t*)^−1^	Estimated
*h*	Reinjection rate of *N* by thymus	0.01 Δ(*t*)^−1^	Estimated
*k*_1_	*E*_*s*_ duplication threshold due to *A*_*s*_	10	Estimated
*k*_2_	*C*-*E*_*s*_ min. killing rate threshold	1	Estimated
*k*_3_	*C*-*E*_*s*_ max. killing rate threshold	50	Estimated
*M*	Number of *N* in safe conditions (leukocyte formula)	196000	in vivo
*C*_0_	Initial number of *C *	180000	in vivo
*p*	No. of injected *E* by treatment administration	760000	in vivo
*q*	No. of injected *Ab* by treatment administration	1000000	in vivo
*τ*	Delay value	1	in vivo
